# Antenatal Opioid Exposure and Cerebral Cortical Maturation in Newborns

**DOI:** 10.1001/jamanetworkopen.2026.14115

**Published:** 2026-05-22

**Authors:** Yao Wu, Stephanie L. Merhar, Carla M. Bann, Jamie E. Newman, Kushal Kapse, Josepheen De Asis-Cruz, Jonathan M. Davis, Namasivayam Ambalavanan, Sara B. De Mauro, Scott A. Lorch, Deanne Wilson-Costello, Brenda B. Poindexter, Nicole Mack, Myriam Peralta-Carcelen, Catherine Limperopoulos

**Affiliations:** 1 Developing Brain Institute, Children’s National Hospital, Washington, DC; 2 Perinatal Institute, Division of Neonatology, Cincinnati Children’s Hospital Medical Center, Cincinnati, Ohio; 3 Department of Pediatrics, University of Cincinnati, Cincinnati, Ohio; 4 Analytics Division, RTI International, Research Triangle Park, North Carolina; 5 Division of General Pediatrics, Tufts Medical Center, Boston, Massachusetts; 6 Department of Pediatrics, University of Alabama at Birmingham, Birmingham; 7 Division of Neonatology, Children’s Hospital of Philadelphia, Philadelphia, Pennsylvania; 8 Department of Pediatrics, University of Pennsylvania Perelman School of Medicine, Philadelphia; 9 Department of Pediatrics, Case Western Reserve University, Cleveland, Ohio; 10 Department of Pediatrics, Emory University, Atlanta, Georgia

## Abstract

**Importance:**

Antenatal opioid exposure is associated with adverse neurodevelopmental outcomes and smaller brain volumes, but the effects of opioids on newborn cortical folding maturation have not been defined. The Advancing Clinical Trials in Neonatal Opioid Withdrawal Outcomes of Babies With Opioid Exposure (OBOE) Study is a multisite prospective longitudinal cohort study examining the association of antenatal opioid exposure with brain maturation and outcomes in newborns.

**Objective:**

To compare cerebral cortical folding in newborns exposed to opioids vs nonexposed controls.

**Design, Setting, and Participants:**

In this cohort study, full-term newborns from the OBOE study with antenatal opioid exposure and nonexposed controls were recruited at 4 US sites, including obstetric clinics, maternal substance use treatment programs, and birth hospitals, from August 5, 2020, to December 28, 2023. Data analysis was performed from August 19, 2020, to March 25, 2026.

**Exposure:**

Newborn opioid exposure, including opioid-only and polysubstance exposure as well as exposure to specific opioids.

**Main Outcomes and Measures:**

Nonsedated T2-weighted magnetic resonance imaging (MRI) data were acquired via harmonized protocols, and 3D brain images were segmented and parcellated using the Developing Brain Region Annotation With Expectation–Maximization pipeline. The inner cortical gray matter surface was used to measure cortical folding across the frontal, parietal, temporal, and occipital lobes. Group differences between opioid-exposed and nonexposed newborns were compared via analysis of covariance, adjusting for postmenstrual age at MRI, sex, birth weight, maternal age, smoking status, and education level.

**Results:**

A total of 259 newborns (mean [SD] gestational age at birth, 39.1 [1.0] weeks; 145 [56.0%] male) were included in the analysis, of whom 164 had antenatal exposure to opioids and 95 were nonexposed controls (mean [SD] postmenstrual age at MRI, 42.8 [2.2] and 42.9 [2.0] weeks, respectively). Compared with nonexposed controls, newborns who had been exposed to opioids had significantly decreased sulcal depth in the frontal (difference, −0.11 mm [95% CI, −0.20 to −0.02 mm]), parietal (difference, −0.19 mm [95% CI, −0.31 to −0.07 mm]), and global (difference, −0.09 mm [95% CI, −0.18 to −0.01 mm]) regions, as well as decreased surface area in the frontal (difference, −1048 mm^2^ [95% CI, −1497 to −598 mm^2^]), parietal (difference, −501 mm^2^ [95% CI, −834 to −168 mm^2^]), temporal (difference, −422 mm^2^ [95% CI, −682 to −162 mm^2^]), occipital (difference, −232 mm^2^ [95% CI, −439 to −26 mm^2^]), and global (difference, −2185 mm^2^ [95% CI, −3327 to −1043 mm^2^]) surfaces. Compared with controls, newborns exposed to methadone showed larger reductions in frontal, parietal, and global surface areas than those exposed to buprenorphine, with parietal surface area significantly reduced only in the methadone-exposed group (difference, −656 mm^2^ [95% CI, −1111 to −202 mm^2^]). Newborns with polysubstance exposure had significantly reduced sulcal depth in the frontal, parietal, and global surfaces, as well as reduced surface area across all lobes compared with controls, whereas opioid-only exposed newborns showed fewer significant differences from controls, with reduced parietal sulcal depth and decreased frontal and global surface areas.

**Conclusions and Relevance:**

In this cohort study, newborns with antenatal exposure to opioids had reduced cerebral cortical sulcal depth and surface area compared with nonexposed controls, with greater reductions among newborns exposed to methadone compared with those exposed to buprenorphine, and in newborns with polysubstance exposure compared with those with opioid exposure only. Ongoing serial MRI and long-term follow-up are under way to assess the impact of these early cortical maturational differences on later neurodevelopment and behavior.

## Key Points

**Question:**

Does cerebral cortical folding differ in newborns who are exposed to opioids antenatally compared with those who are nonexposed?

**Findings:**

In this cohort study of 259 newborns in the US, those exposed to opioids antenatally demonstrated significantly reduced cortical sulcal depth in the frontal, parietal, and global surfaces, as well as decreased cortical surface area across frontal, parietal, temporal, occipital, and global regions Compared with controls, newborns exposed to methadone showed greater surface area reductions than those exposed to buprenorphine; newborns with polysubstance exposure showed greater decreases in sulcal depth and surface area than those exposed only to opioids.

**Meaning:**

These findings suggest that antenatal opioid exposure in newborns is associated with altered neonatal cortical folding, with the extent varying by type of opioid and presence of coexposures.

## Teaser

This cohort study assesses whether antenatal opioid exposure is associated with altered cortical folding in US newborns.

## Introduction

Opioid use during pregnancy is a major public health concern, with serious implications for both pregnant individuals and their children. The recent opioid epidemic in the US led to a 131% increase in pregnant women with opioid use disorder from 2010 to 2017.^1^ In a 2019 study from the US, nearly 7% of pregnant women reported using opioid pain relievers at some point during pregnancy.^2^ This rise in opioid use has been associated with a spectrum of adverse neonatal and neurodevelopmental outcomes. Neonatal opioid withdrawal syndrome and other withdrawal-related complications have been widely reported in opioid-exposed infants.^3^ Importantly, beyond immediate withdrawal symptoms, antenatal opioid exposure is associated with long-term neurodevelopmental impairment in children, including lower cognitive and language abilities,^4,5^ higher rates of attention-deficit/hyperactivity disorder, and deficits in executive functioning.^6^

Emerging neuroimaging evidence indicates that antenatal opioid exposure impairs brain development in offspring. Structural magnetic resonance imaging (MRI) studies in school-aged children have reported reduced volumes in key regions such as the basal ganglia, thalamus, and cerebellar white matter.^7^ Similarly, children in middle-to-late childhood with antenatal exposure to opioid maintenance therapy show reduced total brain volumes and smaller cortical surface areas compared with nonexposed controls.^8^ In adolescents and young adults, antenatal opioid or polysubstance exposure has also been associated with smaller brain volumes, reduced cortical surface areas, and thinner cortex.^9,10^ In newborns, studies have identified smaller volumes in total brain, deep gray matter, thalamic nuclei, insular white matter, subthalamic nuclei, brainstem, and cerebrospinal fluid, as well as enlarged lateral ventricles, in opioid-exposed infants.^11,12^ Our recent large multicenter study^13^ confirmed that opioid-exposed newborns have smaller total and regional brain volumes, with distinct patterns of volume reduction associated with methadone, buprenorphine, and polysubstance exposures.

However, the specific effects of antenatal opioid exposure on cortical morphology, such as cortical gyrification index (ie, the degree of cortical folding) and sulcal depth (ie, the depth of cortical folds), have not been adequately defined. To our knowledge, only 1 fetal MRI study of 14 opioid-exposed and 15 nonexposed fetuses^14^ reported that opioid-exposed fetuses in the third trimester showed reduced cortical gyrification index, sulcal depth, and surface area compared with controls. Altered cortical folding has been associated with conditions such as intellectual disability, epilepsy, autism, and schizophrenia.^15,16,17,18^ Given that cortical folding is a highly dynamic process that accelerates during the third trimester and extends into early postnatal life,^19,20^ antenatal opioid exposure may disrupt this critical developmental process, potentially contributing to long-term cognitive and behavioral impairments.

As part of the Outcomes of Babies With Opioid Exposure (OBOE) study,^21^ investigators from our group used MRI to assess cerebral cortical folding in full-term newborns with and without antenatal opioid exposure. The present study aimed to determine whether antenatal opioid exposure is associated with altered cortical folding in newborns. Given prior evidence that antenatal exposure to different opioid types and coexposures may be associated with differential brain development in offspring,^9,10,13^ secondary analyses were performed to evaluate the association of methadone vs buprenorphine, as well as polysubstance vs opioid-only exposure, with neonatal cortical folding. We hypothesized that newborns with opioid exposure would exhibit altered cortical folding compared with nonexposed controls.

## Methods

### Study Design

The Advancing Clinical Trials in Neonatal Opioid Withdrawal (ACT-NOW) OBOE study is an ongoing, multisite prospective study of newborns with antenatal opioid exposure and nonexposed controls recruited from 4 sites in the US.^22^ We recruited participants from August 5, 2020, to December 28, 2023. Data analysis was performed from August 19, 2020, to March 25, 2026. The study protocol has been published previously.^21^ Families were recruited prenatally in obstetric clinics or maternal substance use treatment programs or postnatally in the birth hospitals. Exposed newborns were eligible if they had been exposed to opioids in the second and/or third trimester and were born at a gestational age of 37 weeks or later. Control newborns were eligible if born at a gestational age of 37 weeks or later with no known or reported opioid exposure; they were recruited from the same birth hospitals as the exposed newborns and targeted with similar sociodemographic characteristics. Exclusion criteria included chromosomal or congenital anomalies with the potential to affect the central nervous system, 5-minute Apgar score less than 5, any requirement for positive pressure ventilation in the neonatal intensive care unit, inability to return for MRI or follow-up, intrauterine growth restriction to less than the third percentile, or maternal alcohol use of 8 or more drinks per week. The initial study visit was scheduled prior to 8 weeks of age and included a brain MRI examination and caregiver questionnaires. Through a single Institutional Review Board at Cincinnati Children’s Hospital Medical Center, all 4 OBOE clinical sites, the neuroimaging core, and the data coordinating center received approval for human participant research, with written informed consent obtained from all participants. This study followed the Strengthening the Reporting of Observational Studies in Epidemiology (STROBE) reporting guideline for cohort studies.

### Demographic Characteristics

Maternal age and race and ethnicity were determined through review of the maternal electronic health record. Race and ethnicity were categorized as Hispanic, non-Hispanic Black, non-Hispanic White, other (including American Indian or Alaska Native, Asian, and Native Hawaiian or Other Pacific Islander), or unknown; these data were included to assess the demographic diversity of the participants. Maternal educational level was obtained from the newborn’s birth certificate. Neonatal demographic data were obtained from the newborns’ electronic health records.

### Substance Exposure

Newborn opioid exposure was determined from maternal history, maternal urine toxicology results at delivery, and/or results of neonatal urine, meconium, or umbilical cord toxicology screening. During the initial visit, mothers completed a comprehensive questionnaire detailing substance use throughout pregnancy. Opioid-only exposure was defined as antenatal exposure exclusively to opioids, even if multiple opioid types were involved. Polysubstance exposure was defined as antenatal opioid exposure combined with other psychoactive substances or medications, such as selective serotonin reuptake inhibitors (SSRIs), cocaine, tetrahydrocannabinol, benzodiazepines, gabapentin, amphetamines, muscle relaxants, psychostimulants, and/or antipsychotics.

### MRI Acquisition

MRI examinations were performed during natural sleep. T2-weighted images were acquired on 3T scanners with protocols harmonized across the 4 sites. Sites 1 and 2 used 3T scanners with 32-channel head coils (turbo spin echo; repetition time, 2500 milliseconds; echo time, 270/251.65 milliseconds; flip angle, 90°; resolution, 0.982 × 0.982 × 1 mm^3^ [Koninklijke Philips NV]). Sites 3 and 4 used Siemens 3T scanners with 32-channel coils (sampling perfection with application optimized contrast using different flip angle evolution; repetition time, 3200 milliseconds; echo time, 492 milliseconds; flip angle, 90°; resolution, 0.982 × 0.982 × 1 mm^3^ for site 3 and 1 × 1 × 1 mm^3^ for site 4 [Siemens Healthineers AG]).

### Image Processing

T2-weighted images were segmented using an automated tool validated for neonatal brain segmentation (Developing Brain Region Annotation With Expectation–Maximization [Draw-EM]).^23^ Results were visually inspected and manually corrected (Figure) in ITK-SNAP software (Penn Image Computing and Science Laboratory at the University of Pennsylvania) by an experienced rater using all 3 planes (axial, coronal, and sagittal). Forty scans were randomly chosen and corrected by a second experienced rater. Interrater reliability using intraclass correlation coefficient for cortical gray matter was 0.96. Raters were blinded to exposure status and to each other’s corrections.

**Figure.**
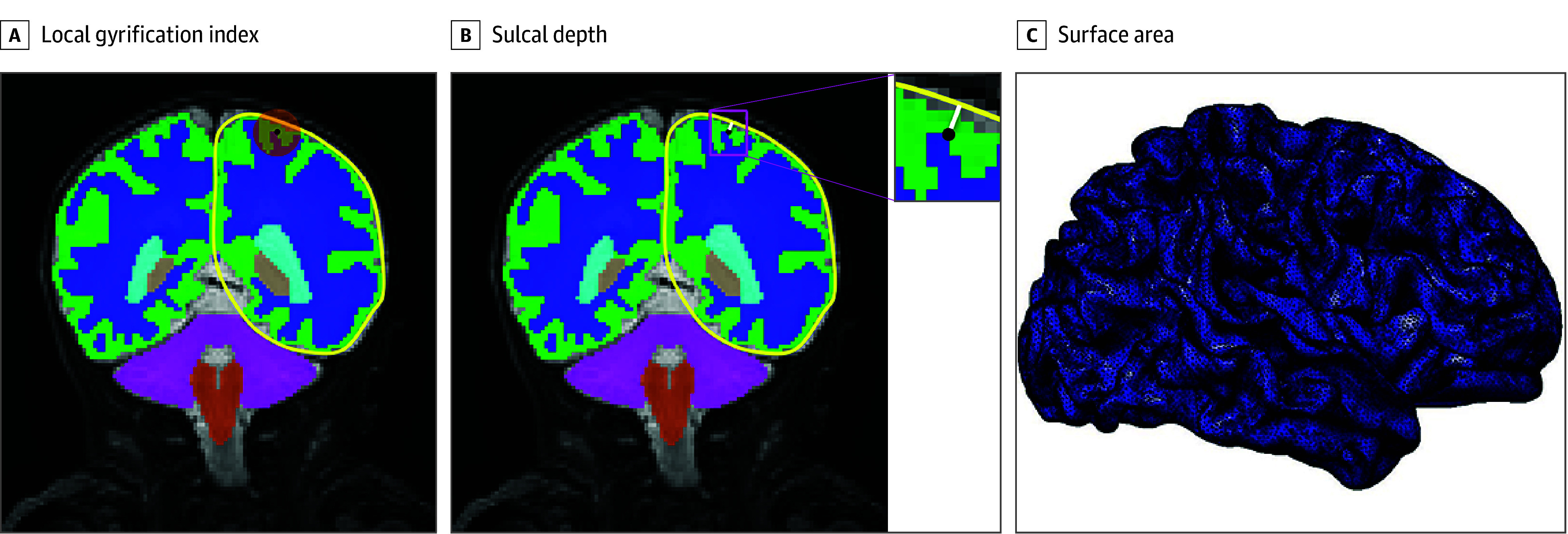
Illustration of Cerebral Cortical Folding Measures on a Newborn Brain at 40.3 Postmenstrual Weeks of Age A, Local gyrification index is calculated as the ratio between the cortical surface area (inner surface of cortical gray matter) and the corresponding area on cerebral hull surface (yellow line) in a sphere centered at each surface vertex (black dot). B, Sulcal depth is calculated as the distance (white line) from each vertex on the cortical surface to the nearest point on the cerebral hull surface. C, Surface area is calculated as the summation of the areas formed by the triangular surface meshes.

After brain segmentation, the inner surface of cerebral cortical gray matter (ie, the border of cortical gray matter and white matter) was used to measure cortical folding.^24^ Frontal, parietal, temporal, and occipital lobes were obtained by consolidating 50 Draw-EM parcellated regions, with visual inspection and manual correction using ITK-SNAP. Measures analyzed for each lobe included (1) local gyrification index: the ratio between the cortical surface area and the corresponding area on cerebral hull surface within a sphere centered at each surface vertex (Figure)^25^; (2) sulcal depth: the distance from each vertex on the cortical surface to the nearest point on the cerebral hull surface^26^; and (3) surface area: the sum of all triangular surface mesh areas.^27^

### Statistical Analysis

Statistical analyses were performed using MATLAB, version R2024a (The MathWorks Inc), and SAS OnDemand for Academics (SAS Institute Inc). Baseline and clinical characteristics of participants in the opioid-exposed and nonexposed groups were compared using independent samples *t* tests or Wilcoxon rank sum tests for continuous variables and Fisher exact tests for categorical variables. Cerebral cortical folding measures in the opioid-exposed and unexposed groups were compared using analysis of covariance (ANCOVA), adjusting for postmenstrual age at MRI, sex, birth weight, maternal age, maternal smoking status, and maternal educational level. Cortical folding measures were compared using ANCOVA with pairwise comparisons among 3 groups (nonexposed, methadone-exposed, and buprenorphine-exposed) as well as among nonexposed, opioid-exposed only, and polysubstance-exposed groups, adjusting for postmenstrual age at MRI, sex, birth weight, maternal age, maternal smoking status, and maternal educational level. *P* values were adjusted for multiple testing based on the false discovery rate according to the Benjamini-Hochberg method.^28^ Two-sided adjusted *P* < .05 was considered statistically significant.

## Results

### Participant Characteristics

The analysis included MRI scans from 259 newborns (mean [SD] gestational age at birth, 39.1 [1.0] weeks; 114 [44.0%] females and 145 [56.0%] males), of whom 164 were exposed to opioids (mean [SD] postmenstrual age at MRI, 42.8 [2.2] weeks) and 95 were nonexposed (mean [SD] postmenstrual age at MRI, 42.9 [2.0] weeks). Participant enrollment is summarized in the eFigure in Supplement 1, and demographic characteristics are shown in Table 1 (sex-specific data are given in eTable 1 in Supplement 1). Compared with controls, newborns in the opioid-exposed group had lower birth weight (mean [SD], 3.2 [0.4] kg vs 3.4 [0.4] kg; *P* < .001) and head circumference at birth (mean [SD], 34.0 [1.4] vs 34.7 [1.3] cm; *P* < .001). Maternal age was higher in mothers of newborns exposed to opioids compared with mothers of nonexposed controls (mean [SD], 30.4 [4.3] vs 28.3 [5.5] years; *P* = .001), and smoking was more common (127 [77.4%] vs 22 [23.2%]; *P* < .001). Maternal educational level also differed between groups, with a higher proportion of mothers in the opioid-exposed group having less than a high school education (38 [23.2%] vs 7 [7.4%]) and fewer having a college or graduate degree (12 [7.3%] vs 27 [28.4%]) compared with controls (*P* < .001). The present study had a smaller sample size than the prior volumetric study of the same cohort^13^ because it applied additional quality control for cortical surface reconstruction and parcellation. The excluded scans due to insufficient data quality are shown in the eFigure in Supplement 1.

**Table 1.** Characteristics of the Study Cohort

Characteristic	Newborn group		*P* value^a^
	Nonexposed (n = 95)	Opioid-exposed (n = 164)	
Maternal age, mean (SD), y	28.3 (5.5)	30.4 (4.3)	.001
Maternal smoking during pregnancy, No. (%)	22 (23.2)	127 (77.4)	<.001
Birth weight, mean (SD), kg	3.4 (0.4)	3.2 (0.4)	<.001
Gestational age at birth, mean (SD), wk	39.3 (1.0)	39.0 (1.0)	.08
Head circumference at birth, mean (SD), cm	34.7 (1.3)	34.0 (1.4)	<.001
Newborn sex			
Female	39 (41.1)	75 (45.7)	.52
Male	56 (58.9)	89 (54.3)	
Vaginal delivery, No. (%)	60 (63.2)	108 (65.9)	.69
1-minute Apgar scores at birth, median (IQR)	8 (8-8)	8 (8-8)	.48
5-minute Apgar scores at birth, median (IQR)	9 (9-9)	9 (9-9)	.30
Postmenstrual age at MRI, mean (SD), wk	42.9 (2.0)	42.8 (2.2)	.78
Maternal race and ethnicity, No. (%)			
Hispanic	3 (3.2)	2 (1.2)	.15
Non-Hispanic Black	21 (22.1)	25 (15.2)	
Non-Hispanic White	68 (71.6)	135 (82.3)	
Other^b^	1 (1.1)	0	
Unknown	2 (2.1)	2 (1.2)	
Maternal educational level			
Less than high school diploma	7 (7.4)	38 (23.2)	<.001
High school graduate	32 (33.7)	62 (37.8)	
Partial college or specialized training	29 (30.5)	46 (28.0)	
College or graduate degree	27 (28.4)	12 (7.3)	
Unknown	0	6 (3.7)	

Abbreviation: MRI, magnetic resonance imaging.

^a^ Differences between opioid-exposed and nonexposed groups were calculated using independent samples *t* tests or Wilcoxon rank sum tests for continuous variables and Fisher exact tests for categorical variables.

^b^ Includes American Indian or Alaska Native, Asian, or Native Hawaiian or Other Pacific Islander.

### Medications Used During Pregnancy

eTable 2 in Supplement 1 lists the medications used during pregnancy, including buprenorphine, methadone, oxycodone, gabapentin, SSRIs, benzodiazepines, amphetamines, fentanyl, antipsychotics, muscle relaxants, psychostimulants, hydrocodone, tetrahydrocannabinol, and others. Buprenorphine (112 [68.3%]) and methadone (42 [25.6%]) were the most commonly used opioids.

### Cortical Folding in Newborns Exposed to Opioids vs Nonexposed Controls

Among newborns exposed to opioids, sulcal depth was reduced in the frontal (3.24 mm [95% CI, 3.13-3.35 mm] vs 3.13 mm [95% CI, 3.06-3.21 mm]; difference, −0.11 mm [95% CI, −0.20 to −0.02 mm]; adjusted *P* = .05) and parietal (4.51 mm [95% CI, 4.37-4.65 mm] vs 4.30 mm [95% CI, 4.20-4.40 mm]; difference, −0.19 mm [95% CI, −0.31 to −0.07 mm]; adjusted *P* = .003) lobes, as well as the global surface (3.51 mm [95% CI, 3.41-3.61 mm] vs 3.41 mm [95% CI, 3.34-3.48 mm]; difference, −0.09 mm [95% CI, −0.18 to −0.01 mm]; adjusted *P* = .05) compared with nonexposed controls (Table 2; sex-specific data are given in eTable 3 in Supplement 1). Cortical surface area was also reduced in the frontal (10 988 mm^2^ [95% CI, 10 441-11 535 mm^2^] vs 9959 mm^2^ [95% CI, 9576-10 341 mm^2^]; difference, −1048 mm^2^ [95% CI, −1497 to −598 mm^2^]; adjusted *P* < .001), parietal (7230 mm^2^ [95% CI, 6827-7633 mm^2^] vs 6739 mm^2^ [95% CI, 6457-7022 mm^2^]; difference, −501 mm^2^ [95% CI, −834 to −168 mm^2^]; adjusted *P* = .01), temporal (6452 mm^2^ [95% CI, 6136-6769 mm^2^] vs 6050 mm^2^ [95% CI, 5828-6273mm^2^]; difference, −422 mm^2^ [95% CI, −682 to −162 mm^2^]; adjusted *P* = .01), and occipital (3800 mm^2^ [95% CI, 3549-4051 mm^2^] vs 3575 mm^2^ [95% CI, 3399-3750 mm^2^]; difference, −232 mm^2^ [95% CI, −439 to −26 mm^2^]; adjusted *P* = .05) lobes as well as the global surface (28 498 mm^2^ [95% CI, 27 109-29 888 mm^2^] vs 26 385 mm^2^ [95% CI, 25 416-27 353 mm^2^]; difference, −2185 mm^2^ [95% CI, −3327 to −1043 mm^2^]; adjusted *P* = .003) in opioid-exposed newborns (Table 2).

**Table 2.** Comparison of Cerebral Cortical Folding in Opioid-Exposed vs Nonexposed Newborns

Measure	Newborn group, least-squares mean (95% CI)^a^			Adjusted *P* value^b^
	Nonexposed (n = 95)	Opioid exposed (n = 164)	Difference (95% CI)	
**Local gyrification index**				
Frontal	1.91 (1.86 to 1.97)	1.88 (1.84 to 1.92)	−0.03 (−0.08 to 0.01)	.25
Parietal	2.48 (2.38 to 2.58)	2.44 (2.37 to 2.51)	−0.03 (−0.12 to 0.05)	.46
Temporal	1.95 (1.89 to 2.01)	1.95 (1.91 to 2.00)	0.00 (−0.05 to 0.05)	.88
Occipital	2.05 (1.97 to 2.14)	2.06 (2.00 to 2.12)	0.01 (−0.06 to 0.08)	.83
Global	2.09 (2.02 to 2.15)	2.07 (2.02 to 2.11)	−0.02 (−0.07 to 0.04)	.63
**Sulcal depth, mm**				
Frontal	3.24 (3.13 to 3.35)	3.13 (3.06 to 3.21)	−0.11 (−0.20 to −0.02)	.05
Parietal	4.51 (4.37 to 4.65)	4.30 (4.20 to 4.40)	−0.19 (−0.31 to −0.07)	.003
Temporal	3.06 (2.94 to 3.17)	3.03 (2.95 to 3.11)	−0.02 (−0.11 to 0.07)	.75
Occipital	3.16 (3.03 to 3.29)	3.10 (3.01 to 3.19)	−0.05 (−0.16 to 0.06)	.45
Global	3.51 (3.41 to 3.61)	3.41 (3.34 to 3.48)	−0.09 (−0.18 to −0.01)	.05
**Surface area, mm^2^**				
Frontal	10 988 (10 441 to 11 535)	9959 (9576 to 10 341)	−1048 (−1497 to −598)	<.001
Parietal	7230 (6827 to 7633)	6739 (6457 to 7022)	−501 (−834 to −168)	.01
Temporal	6452 (6136 to 6769)	6050 (5828 to 6273)	−422 (−682 to −162)	.01
Occipital	3800 (3549 to 4051)	3575 (3399 to 3750)	−232 (−439 to −26)	.05
Global	28 498 (27 109 to 29 888)	26 385 (25 416 to 27 353)	−2185 (−3327 to −1043)	.003

^a^ Results were derived from analysis of covariance, controlling for postmenstrual age at magnetic resonance imaging scan, sex, birth weight, maternal age, maternal smoking status, and maternal educational level.

^b^ Adjusted for multiple testing based on the false discovery rate according to the Benjamini-Hochberg method. Adjusted *P* < .05 was considered statistically significant.

### Cortical Folding in Newborns Exposed to Different Opioid Types

Table 3 gives pairwise comparisons of cerebral cortical folding among newborns exposed to methadone, buprenorphine, and nonexposed controls (sex-specific data are given in eTable 4 in Supplement 1). Compared with controls, newborns showed significantly reduced parietal sulcal depth among those exposed to methadone (4.27 mm [95% CI, 4.12-4.42 mm] vs 4.47 mm [95% CI, 4.33-4.62 mm]; difference, −0.20 mm [95% CI, −0.37 to −0.04 mm]; adjusted *P* = .05) or buprenorphine (4.31 mm [95% CI, 4.21-4.42 mm]; difference, −0.16 mm [95% CI, −0.29 to −0.03 mm]; adjusted *P* = .05). Cortical surface area was also reduced in frontal, temporal, and global surfaces in newborns exposed to methadone or buprenorphine, compared with controls (Table 3).

**Table 3.** Comparison of Cerebral Cortical Folding in Newborns Antenatally Exposed to Different Opioid Types

Measure	Newborn group, least-squares mean (95% CI)^a^		
	Nonexposed (n = 95)	Methadone exposed (n = 37)	Buprenorphine exposed (n = 108)
**Local gyrification index**			
Frontal	1.91 (1.85-1.96)	1.83 (1.78-1.89)	1.88 (1.84-1.92)
Parietal	2.46 (2.36-2.56)	2.38 (2.28-2.48)	2.45 (2.38-2.52)
Temporal	1.93 (1.87-2.00)	1.93 (1.86-2.00)	1.96 (1.91-2.00)
Occipital	2.04 (1.96-2.13)	2.03 (1.95-2.12)	2.06 (2.00-2.12)
Global	2.07 (2.00-2.14)	2.02 (1.95-2.09)	2.07 (2.02-2.12)
**Sulcal depth, mm**			
Frontal	3.23 (3.11-3.34)	3.11 (2.99-3.23)	3.12 (3.04-3.21)
Parietal	4.47 (4.33-4.62)	4.27 (4.12-4.42)^b^	4.31 (4.21-4.42)^b^
Temporal	3.02 (2.90-3.14)	3.02 (2.90-3.14)	3.04 (2.95-3.12)
Occipital	3.18 (3.05-3.31)	3.08 (2.95-3.22)	3.09 (2.99-3.18)
Global	3.49 (3.39-3.59)	3.40 (3.29-3.50)	3.41 (3.34-3.49)
**Surface area, mm^2^**			
Frontal	10 870 (10 333-11 406)	9482 (8935-10 030)^c^	9980 (9590-10 369)^d^
Parietal	7133 (6729-7538)	6477 (6064-6890)^b^	6756 (6460-7052)
Temporal	6401 (6085-6717)	5925 (5600-6249)^b^	6048 (5817-6279)^b^
Occipital	3754 (3497-4011)	3443 (3180-3705)	3590 (3404-3777)
Global	28 184 (26 805-29 564)	25 273 (23 866-26 680)^d^	26 478 (25 479-27 478)^b^

^a^ Results were derived from analysis of covariance, controlling for postmenstrual age at magnetic resonance imaging scan, sex, birth weight, maternal age, maternal smoking status, and maternal educational level. Pairwise comparisons were performed among nonexposed, methadone-exposed, and buprenorphine-exposed groups. Adjusting for multiple testing was based on the false discovery rate according to the Benjamini-Hochberg method. Adjusted *P* < .05 was considered statistically significant.

^b^ Significantly different from nonexposed controls (adjusted *P* < .05).

^c^ Significantly different from nonexposed controls (adjusted *P* < .001).

^d^ Significantly different from nonexposed controls (adjusted *P* < .01).

The reduction in parietal surface area was significant only among newborns with methadone exposure compared with controls (6477 mm^2^ [95% CI, 6064-6890 mm^2^] vs 7133 mm^2^ [95% CI, 6729-7538 mm^2^]; difference, −656 mm^2^ [95% CI, −1111 to −202 mm^2^]; adjusted *P* = .04), but not among those exposed to buprenorphine compared with controls (6756 mm^2^ [95% CI, 6460-7052 mm^2^] vs 7133 mm^2^ [95% CI, 6729-7538 mm^2^]; difference, −377 mm^2^ [95% CI, −741 to −14] mm^2^]; adjusted *P* = .15) (Table 3). Moreover, when compared with controls, newborns with methadone exposure showed greater reductions in frontal surface area (9482 mm^2^ [95% CI, 8935-10 030 mm^2^] vs 10 870 mm^2^ [95% CI, 10 333-11 406 mm^2^]; difference, −1387 mm^2^ [95% CI, −1990 to −784 mm^2^]; adjusted *P* < .001) than buprenorphine-exposed newborns (9980 mm^2^ [95% CI, 9590-10 369 mm^2^]; difference, −890 mm^2^ [95% CI, −1371 to −409 mm^2^]; adjusted *P* = .004). Newborns with methadone exposure also showed greater reductions in surface area of global regions compared with controls (25 273 mm^2^ [95% CI, 23 866-26 680 mm^2^] vs 28 184 mm^2^ [95% CI, 26 805-29 564 mm^2^]; difference, −2912 mm^2^ [95% CI, −4461 to −1362 mm^2^]; adjusted *P* = .005) than newborns with buprenorphine exposure (26 478 mm^2^ [95% CI, 25 479-27 478 mm^2^]; difference, −1706 mm^2^ [95% CI, −2943 to −469 mm^2^]; adjusted *P* = .05) regions. No significant differences in cortical folding measures were found between the methadone- and buprenorphine-exposed groups.

### Cortical Folding Differences in Newborns With Opioid-Only and Polysubstance Exposure vs Nonexposed Controls

Given that newborns with opioid exposure showed impaired sulcal depth and surface area compared with nonexposed controls (Table 2), we conducted a subgroup analysis with pairwise comparisons of cerebral sulcal depth and surface area among newborns with opioid-only exposure and polysubstance exposure (opioids plus additional substances) and nonexposed controls (Table 4; sex-specific data are given in eTable 5 in Supplement 1). Compared with nonexposed controls, the polysubstance exposure group showed significant reductions in sulcal depth in the frontal (3.11 mm [95% CI, 3.03-3.19 mm] vs 3.25 mm [95% CI, 3.14-3.36 mm]; difference, −0.14 mm [95% CI, −0.24 to −0.04 mm]; adjusted *P* = .02), parietal (4.29 mm [95% CI, 4.19-4.39 mm] vs 4.52 mm [95% CI, 4.37-4.66 mm]; difference, −0.23 mm [95% CI, −0.36 to −0.10 mm]; adjusted *P* = .006), and global (3.40 mm [95% CI, 3.32-3.47 mm] vs 3.52 mm [95% CI, 3.42-3.62 mm]; difference, −0.12 mm [95% CI, −0.21 to −0.03 mm]; adjusted *P* = .02) surfaces. Surface area was also significantly reduced in the polysubstance group across the frontal (9968 mm^2^ [95% CI, 9566-10 370 mm^2^] vs 10 983 mm^2^ [95% CI, 10 432-11 535 mm^2^]; difference, −1015 mm^2^ [95% CI, −1516 to −514 mm^2^]; adjusted *P* = .002), parietal (6710 mm^2^ [95% CI, 6412-7008 mm^2^] vs 7244 mm^2^ [95% CI, 6838-7650 mm^2^]; difference, −534 mm^2^ [95% CI, −903 to −164 mm^2^]; adjusted *P* = .02), temporal (6005 mm^2^ [95% CI, 5771-6238 mm^2^] vs 6474 mm^2^ [95% CI, 6156-6792 mm^2^]; difference, −469 mm^2^ [95% CI, −759 to −180 mm^2^]; adjusted *P* = .006), and occipital (3552 mm^2^ [95% CI, 3368-3737 mm^2^] vs 3810 mm^2^ [95% CI, 3558-4063 mm^2^]; difference, −258 mm^2^ [95% CI, −488 to −28 mm^2^]; adjusted *P* = .05) lobes, as well as the global surface (26 339 mm^2^ [95% CI, 25 320-27 358 mm^2^] vs 28 521 mm^2^ [95% CI, 27 120-29 922 mm^2^]; difference, −2182 mm^2^ [95% CI, −3454 to −910 mm^2^]; adjusted *P* = .006).

**Table 4.** Comparison of Cerebral Sulcal Depth and Surface Area Among Newborns Exposed to Opioids Only or Opioids Plus Additional Substances and Nonexposed Controls

Measure	Newborn group, least-squares mean (95% CI)^a^		
	Nonexposed (n = 95)	Opioids only (n = 45)	Opioids plus other substances (n = 119)^b^
**Sulcal depth, mm**			
Frontal	3.25 (3.14-3.36)	3.20 (3.09-3.31)	3.11 (3.03-3.19)^c^
Parietal	4.52 (4.37-4.66)	4.34 (4.20-4.48)^c^	4.29 (4.19-4.39)^d^
Temporal	3.07 (2.95-3.18)	3.10 (2.99-3.21)	3.01 (2.93-3.10)
Occipital	3.15 (3.02-3.29)	3.07 (2.94-3.21)	3.11 (3.01-3.20)
Global	3.52 (3.42-3.62)	3.47 (3.37-3.56)	3.40 (3.32-3.47)^c^
**Surface area, mm^2^**			
Frontal	10 983 (10 432-11 535)	9927 (9381-10 474)^d^	9968 (9566-10 370)^d^
Parietal	7244 (6838-7650)	6830 (6428-7232)	6710 (6412-7008)^c^
Temporal	6474 (6156-6792)	6193 (5876-6510)	6005 (5771-6238)^d^
Occipital	3810 (3558-4063)	3645 (3394-3895)	3552 (3368-3737)^b^
Global	28 521 (27 120-29 922)	26 529 (25 143-27 915)^c^	26 339 (25 320-27 358)^d^

^a^ Results were derived from analysis of covariance, controlling for postmenstrual age at magnetic resonance imaging scan, sex, birth weight, maternal age, maternal smoking status, and maternal educational level. Pairwise comparisons were performed among nonexposed newborns, those exposed to opioids only, and those exposed to polysubstances. Adjusting for multiple testing was based on the false discovery rate according to the Benjamini-Hochberg method. Adjusted *P* < .05 was considered statistically significant.

^b^ Other substances include cocaine, benzodiazepines, selective serotonin reuptake inhibitors, amphetamines, gabapentin, psychostimulants, muscle relaxants, tetrahydrocannabinol, and antipsychotics.

^c^ Significantly different from nonexposed controls (adjusted *P* < .05).

^d^ Significantly different from nonexposed controls (adjusted *P* < .01).

In comparison, the group with opioid-only exposure showed fewer significant differences when compared with controls, with reductions observed in parietal sulcal depth (4.34 mm [95% CI, 4.20-4.48 mm] vs 4.52 [95% CI, 4.37-4.66 mm]; difference, −0.18 mm [95% CI, −0.33 to −0.03 mm]; adjusted *P* = .03) and frontal (9927 mm^2^ [95% CI, 9381-10 474 mm^2^] vs 10 983 [95% CI, 10 432-11 535 mm^2^]; difference, −1056 mm^2^ [95% CI, −1627 to −485 mm^2^]; adjusted *P* = .004) and global surface areas (26 529 mm^2^ [95% CI, 25 143-27 915 mm^2^] vs 28 521 mm^2^ [95% CI, 27 120-29 922 mm^2^]; difference, −1992 [95% CI, −3441 to −542 mm^2^]; adjusted *P* = .02). No significant differences in sulcal depth or surface area were found between the opioid-only and polysubstance exposure groups.

## Discussion

In this large, multisite cohort study, we found that antenatal opioid exposure was associated with impaired cerebral cortical folding in newborns. Newborns who had been exposed to opioids showed decreased sulcal depth in the frontal, parietal, and global regions, as well as reduced surface area in the frontal, parietal, temporal, occipital, and global surfaces. We also demonstrated that when compared with controls, newborns exposed to methadone had greater reductions in frontal, parietal, and global surface area than newborns exposed to buprenorphine. Furthermore, newborns with polysubstance exposure showed more significant reductions in sulcal depth and surface area than newborns with opioid-only exposure when compared with controls. Our findings of impaired regional cerebral cortical folding in newborns with antenatal opioid exposure may serve as early biomarkers of later neurodevelopmental dysfunction in this high-risk population.

Cerebral cortical folding begins in the second trimester and accelerates rapidly during the third trimester.^19,29^ Reduced sulcal depth and surface area may indicate disrupted cellular and molecular processes underlying cortical maturation. Antenatal opioid exposure may impair cortical folding through several mechanisms, such as neuroinflammation,^30,31,32^ impaired neuronal growth,^33^ and disrupted myelination.^34,35^ Opioids can cross the placenta and interfere with multiple neurodevelopmental pathways. Inflammation has been associated with disturbances in cortical growth and connectivity.^36^ In animal models, antenatal methadone exposure induces widespread microstructural alterations, including increased diffusivity and reduced dendritic complexity, in cortical and subcortical regions that persist into early adulthood.^33^ Additional animal studies suggest that antenatal exposure to buprenorphine or methadone disrupts myelination,^34,35^ while opioids may also induce neuronal apoptosis and reduce neurogenesis.^33^ These mechanisms could reduce cortical neurons and impair cortical plate expansion, limiting the mechanical forces necessary for normal gyral and sulcal development. Given that cortical folding accelerates during late gestation and continues into the early postnatal period, this stage likely represents a critical window of vulnerability to opioid-related neurotoxic effects.

To our knowledge, our study is the first to quantify cortical folding alterations during the early postnatal period. We identified reduced sulcal depth in the frontal and parietal lobes, as well as smaller surface area in the frontal, parietal, temporal, and occipital lobes, reflecting region-specific differences in cortical development. Early sulci such as the Sylvian fissure form at 13 to 17 weeks of gestation, followed by periinsular sulci at 18 to 19 weeks and the central sulcus at 20 to 22 weeks. Folding and surface expansion progress at different rates; the fastest progression occurs in the posterior temporal and parietal lobes, particularly between 25 and 30 weeks, and continues rapidly in all lobes through the third trimester into the early postnatal period.^37,38,39^ Functionally, the frontal cortex underlies executive function and behavioral regulation; the parietal lobe supports sensorimotor integration and attention, domains frequently affected in opioid-exposed children; the temporal cortex is involved in memory, auditory, and language processing; and the occipital lobe supports visual processing.^40,41,42,43,44,45,46^ Our findings of reduced surface area and sulcal depth align with our recent report of decreased global brain and regional cortical volumes in opioid-exposed newborns,^13^ suggesting that both measures may reflect delayed cortical maturation. Moreover, the observed reductions in cortical surface area are also consistent with studies in school-aged children exposed antenatally to opioid maintenance therapy^8^ and in adolescents and young adults with antenatal opioid or polysubstance exposure.^9^ Together, these findings suggest that cortical maturation may be disrupted in early life among opioid-exposed newborns, which may represent the earliest structural manifestation of neurodevelopmental vulnerability in offspring with opioid exposure.

We also observed, for the first time, differential effects of methadone and buprenorphine exposure on cortical maturation. Although both were associated with reduced cortical sulcal depth and surface area, methadone-exposed newborns showed more pronounced reductions in surface area in frontal, parietal, and global regions, suggesting a potentially greater neurotoxic effect. Prior clinical studies have reported that buprenorphine exposure is associated with higher gestational age and birth weight^47^ and fewer withdrawal symptoms^48^ compared with methadone exposure. We also found differential effects of these medications on neonatal white matter and amygdala volumes.^13^ These differences may, in part, reflect their pharmacologic distinctions, as buprenorphine is a partial μ-opioid receptor agonist, while methadone is a full agonist.^49,50^ In human cortical organoids, methadone exposure caused growth restriction, whereas buprenorphine did not, possibly due to buprenorphine’s κ-antagonist properties.^51^ In animal models, buprenorphine disrupted interneuron migration and cortical network activity via the nociceptin opioid peptide receptor,^52,53^ which is highly expressed in cortical and limbic regions.^52^ Pharmacokinetic studies in rodents further showed that fetal brain concentrations of methadone are approximately twice those of the maternal brain, whereas buprenorphine concentrations are only one-third of maternal levels.^54^ Other animal studies showed reduced neurogenesis following buprenorphine exposure,^55^ and increased neuroinflammation after methadone exposure.^31^ Together, these findings indicate both medications impact cortical development, although through potentially distinct mechanisms and toxic effects.

Our subanalysis revealed reduced cortical surface area in newborns exposed only to opioids and those with polysubstance exposure, with more pronounced effects in the polysubstance-exposed group. This aligns with prior studies reporting that school-aged children and adolescents with antenatal polysubstance exposure exhibit greater reductions in accumbens, cerebellar cortex, cerebral and cerebellar white matter, and intracranial volumes than those exposed only to opiates (eg, heroin).^9,10^ Polysubstance exposure may compound any effects on the developing brain through overlapping neurotoxic mechanisms. Substances coused with opioids, such as benzodiazepines, tetrahydrocannabinol, and SSRIs, can independently alter neurogenesis, synaptogenesis, or myelination.^56,57,58^ For example, antenatal SSRI exposure has been associated with altered cortical thickness and surface area in children.^59^ Combined exposure may amplify these disruptions through converging inflammatory and apoptotic pathways, further impairing cortical development. The heterogeneity of polysubstance exposure in our cohort limits our ability to isolate the effects of specific substances, but our results underscore the compounded neurodevelopmental risks in this population.^60,61^

### Strengths and Limitations

The strengths of this study include its prospective design, large sample size, inclusion of nonexposed controls, and harmonized multisite imaging protocols. Adjustment for key confounders enhances the validity of our findings. However, the study also has several limitations. First, residual confounding from factors such as maternal stress^62,63^ cannot be ruled out; specifically, maternal stress can be broadly categorized into psychological (eg, depression and anxiety),^63,64^ socioeconomic (eg, poverty and low educational levels),^65,66^ and behavioral (eg, substance use, avoidance behaviors, and relationship conflict) stressors.^67,68^ These factors are common in populations with opioid use,^63,64,65,66,67,68^ and prenatal maternal stress has been associated with altered brain structural and functional outcomes in offspring.^62^ Additional confounders in this population may include trauma history, access to and use of prenatal care, dietary habits, and environmental instability, which were difficult to fully capture and control. Second, we were unable to assess the effects of opioid dose or duration due to the lack of detailed data. The polysubstance-exposed group was heterogeneous, with some newborns only having 1 additional exposure besides opioids and others having multiple additional exposures, and our sample size did not allow for comparison of different substances. Furthermore, cortical folding was analyzed at global and lobar levels rather than at finer regional scales. The Draw-EM–based parcellation is not optimized for within-lobe cortical folding analyses, as it lacks consistent subdivisions across the main cerebral lobes (eg, only the temporal lobe is subdivided, whereas the frontal, parietal, and occipital lobes are not). Future work will incorporate cortical surface-based parcellation approaches to enable more precise characterization of cortical folding at finer regional scales. Finally, this study did not examine the impact of these early cortical impairments on child functional outcomes; follow-up is ongoing to examine their neurodevelopmental and behavioral impact.

## Conclusions

In this cohort study of full-term newborns with antenatal opioid exposure, newborns who had been exposed to opioids showed region-specific impairments in cerebral cortical folding, including reduced frontal, parietal, and global sulcal depth and smaller surface areas in the frontal, parietal, temporal, occipital, and overall cortical regions. The extent of cerebral cortical impairment differed based on the opioid type and presence of polysubstance exposure. Continued longitudinal neuroimaging and neurodevelopmental assessments are currently underway to examine the trajectory of brain development and clinical significance of these early cortical changes, potentially informing early interventions to support neurodevelopment in this vulnerable population.

## Notes

Supplement 1. **eFigure.** Study Flow Diagram **eTable 1.** Sex-Specific Characteristics of the Study Cohort **eTable 2.** Self-Reported Maternal Medication Use During Pregnancy **eTable 3.** Sex-Specific Comparison of Cerebral Cortical Folding in Opioid-Exposed vs Nonexposed Newborns **eTable 4.** Sex-Specific Comparison of Cerebral Cortical Folding in Newborns Antenatally Exposed to Different Opioid Types **eTable 5.** Sex-Specific Comparison of Cerebral Sulcal Depth and Surface Area Among Newborns Exposed to Opioids Only or Opioids Plus Additional Substances and Nonexposed Controls

Supplement 2.
Data Sharing Statement
