# Supplementary material for: Antenatal Opioid Exposure and Cerebral Cortical Maturation in Newborns
Source: JAMA Netw Open. 2026 May 22;9(5):e2614115. doi: 10.1001/jamanetworkopen.2026.14115 (PMC13197868; doi:10.1001/jamanetworkopen.2026.14115)
Supplement: Supplement 1. — eFigure. Study Flow Diagram eTable 1. Sex-Specific Characteristics of the Study Cohort eTable 2. Self-Reported Maternal Medication Use During Pregnancy eTable 3. Sex-Specific Comparison of Cerebral Cortical Folding in Opioid-Exposed vs Nonexposed Newborns eTable 4. Sex-Specific Comparison of Cerebral Cortical Folding in Newborns Antenatally Exposed to Different Opioid Types eTable 5. Sex-Specific Comparison of Cerebral Sulcal Depth and Surface Area Among Newborns Exposed to Opioids Only or Opioids Plus Additional Substances and Nonexposed Controls [file jamanetwopen-e2614115-s001.pdf]

Supplementary Online Content

Wu Y, Merhar SL, Bann CM, et al. Antenatal opioid exposure and cerebral cortical maturation in newborns. *JAMA Netw Open.* 2026;9(5):e2614115. doi:10.1001/jamanetworkopen.2026.14115

eFigure. Study Flow Diagram

eTable 1. Sex-Specific Characteristics of the Study Cohort

eTable 2. Self-Reported Maternal Medication Use During Pregnancy

eTable 3. Sex-Specific Comparison of Cerebral Cortical Folding in Opioid-Exposed vs Nonexposed Newborns

eTable 4. Sex-Specific Comparison of Cerebral Cortical Folding in Newborns Antenatally Exposed to Different Opioid Types

eTable 5. Sex-Specific Comparison of Cerebral Sulcal Depth and Surface Area Among Newborns Exposed to Opioids Only or Opioids Plus Additional Substances and Nonexposed Controls

This supplementary material has been provided by the authors to give readers additional information about their work.

© 2026 Wu Y et al. *JAMA Network Open*.

eFigure. Study Flow Diagram

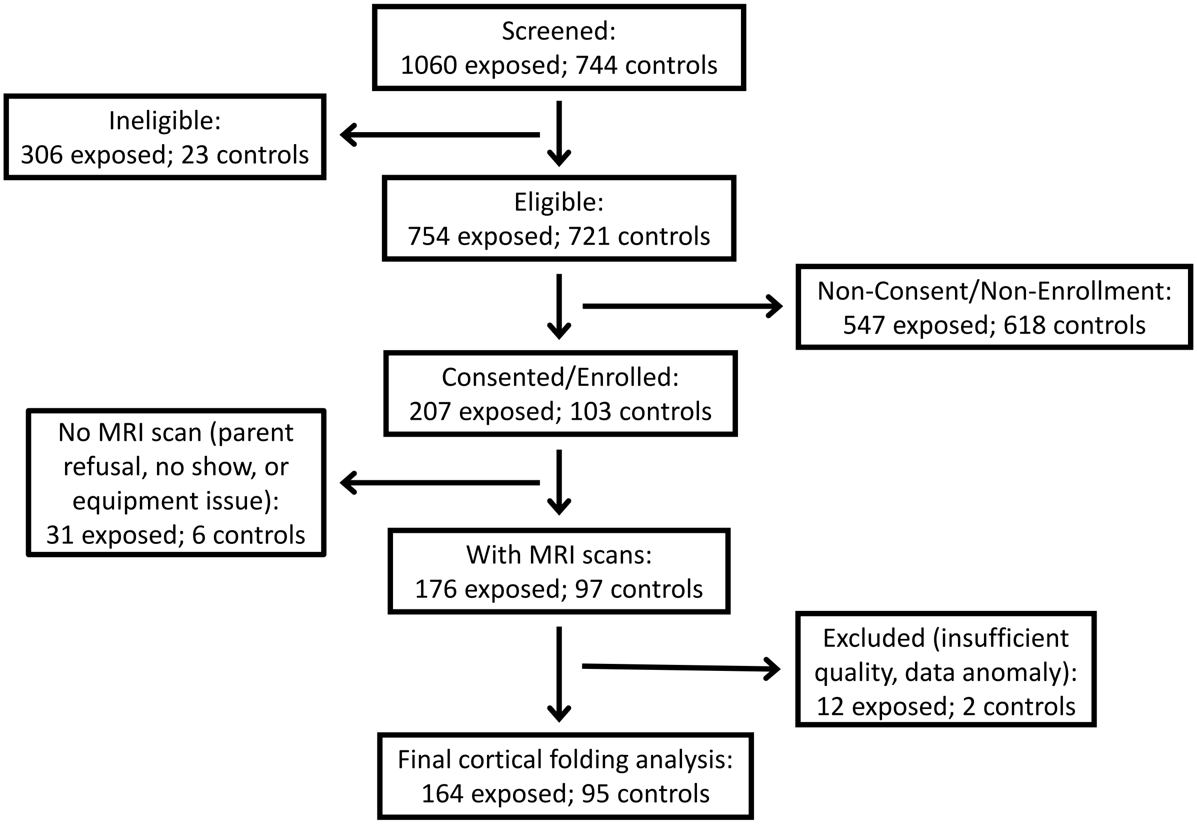

© 2026 Wu Y et al. *JAMA Network Open*.

eTable 1. Sex-Specific Characteristics of the Study Cohort

Characteristic	Newborn group, males/females		*P* value[a]
	Nonexposed (n = 95)	Opioid-exposed (n = 164)	
Maternal age, mean (SD), y	27.8 (4.9)/28.9 (6.2)	30.9 (4.1)/29.8 (4.4)	.001
Maternal smoking during pregnancy, No. (%)	12 (12.6)/10 (10.5)	68 (41.5)/59 (36.0)	<.001
Birth weight, mean (SD), kg	3.5 (0.5)/3.4 (0.4)	3.3 (0.4)/3.1 (0.4)	<.001
Gestational age at birth, mean (SD), wk	39.1 (0.9)/39.5 (1.0)	39.0 (1.0)/39.1 (1.1)	.08
Head circumference at birth, mean (SD), cm	34.9 (1.4)/34.3 (1.3)	34.2 (1.6)/33.8 (1.3)	<.001
Newborn sex			
Male	56 (58.9)	89 (54.3)	.52
Female	39 (41.1)	75 (45.7)	
Vaginal delivery, No. (%)	34 (35.8)/26 (27.4)	54 (32.9)/54 (32.9)	.69
1-minute Apgar scores at birth, median (IQR)	8 (8-8)/8 (8-8)	8 (8-8)/8 (8-8)	.48
5-minute Apgar scores at birth, median (IQR)	9 (9-9)/9 (9-9)	9 (9-9)/9 (9-9)	.30
Postmenstrual age at MRI, mean (SD), wk	42.9 (2.0)/43.0 (2.0)	43.1 (2.1)/42.6 (2.3)	.78
Maternal race and ethnicity, No. (%)			
Hispanic	2 (2.1)/1 (1.1)	2 (1.2)/0	.15
Non-Hispanic Black	12 (12.6)/9 (9.5)	11 (6.7)/14 (8.5)	
Non-Hispanic White	41 (43.2)/27 (28.4)	76 (46.3)/59 (36.0)	
Other[b]	0/1 (1.1)	0/0	
Unknown	1 (1.1)/1 (1.1)	0/2 (1.2)	
Maternal educational level			
Less than high school diploma	3 (3.2)/4 (4.2)	25 (15.3)/13 (7.9)	<.001
High school graduate	21 (22.1)/11 (11.6)	29 (17.7)/33 (20.1)	
Partial college or specialized training	18 (18.9)/11 (11.6)	24 (14.6)/22 (13.4)	
College or graduate degree	14 (14.7)/13 (13.7)	7 (4.3)/5 (3.0)	
Unknown	0/0	4 (2.4)/2 (1.2)	

Abbreviation: MRI, magnetic resonance imaging.

[a]Differences between opioid-exposed and nonexposed groups (overall sample including both males and females) were calculated using independent samples *t* tests or Wilcoxon rank sum tests for continuous variables and Fisher exact tests for categorical variable.

[b]Includes American Indian or Alaska Native, Asian, or Native Hawaiian or Other Pacific Islander.

© 2026 Wu Y et al. *JAMA Network Open*.

eTable 2. Self-Reported Maternal Medication Use During Pregnancy

**N (%) in the overall sample [males/females]**	**Opioid-exposed (n=164)**	**Unexposed (n=95)**
Buprenorphine	112 (68) [62 (38) / 50 (30)]	
Tetrahydrocannabinol	64 (39) [35 (21) / 29 (18)]	3 (3) [1 (1) / 2 (2)]
Methadone	42 (26) [24 (15) / 18 (11)]	
Oxycodone	30 (18) [13 (8) / 17 (10)]	1 (1)* [1 (1) / 0 (0)]
Gabapentin	27 (16) [14 (9) / 13 (8)]	
Selective serotonin reuptake inhibitors	21 (13) [12 (7) / 9 (5)]	16 (17) [11 (12) / 5 (5)]
Benzodiazepines	25 (15) [13 (8) / 12 (7)]	2 (2) [1 (1) / 1 (1)]
Amphetamines	20 (12) [10 (6) / 10 (6)]	
Fentanyl	19 (12) [11 (7) / 8 (5)]	
Antipsychotics	13 (8) [8 (5) / 5 (3)]	
Muscle relaxants[a]	9 (5) [5 (3) / 4 (2)]	4 (4) [4 (4) / 0 (0)]
Psychostimulants[b]	10 (6) [5 (3) / 5 (3)]	
Hydrocodone	7 (4) [3 (2) / 4 (2)]	
Hydromorphone	4 (2) [2 (1) / 2 (1)]	
Heroin	4 (2) [2 (1) / 2 (1)]	
Morphine	2 (1) [1 (1) / 1 (1)]	
Cocaine	2 (1) [1 (1) / 1 (1)]	
Other	23 (14) [14 (9) / 9 (5)]	2 (2) [1 (1) / 1 (1)]

*One mother of a control infant reported minimal oxycodone use after an injury in the second trimester. Maternal urine toxicology screen at delivery and infant umbilical cord toxicology were negative for all substances.

[a] Muscle relaxants included cyclobenzaprine

[b] Psychostimulants included Vyvanse, Adderall, and Ritalin

© 2026 Wu Y et al. *JAMA Network Open*.

eTable 3. Sex-Specific Comparison of Cerebral Cortical Folding in Opioid-Exposed vs Nonexposed Newborns

Measure	Newborn group, least-squares mean (95% CI) males/females[a]		Adjusted *P* value[b]
	Nonexposed (n = 95)	Opioid exposed (n = 164)	
**Local gyrification index**			
Frontal	1.93 (1.87 to 1.98)/1.90 (1.85 to 1.96)	1.89 (1.85 to 1.93)/1.87 (1.83 to 1.91)	.25
Parietal	2.50 (2.40 to 2.60)/2.46 (2.37 to 2.56)	2.46 (2.39 to 2.53)/2.42 (2.36 to 2.49)	.46
Temporal	1.97 (1.91 to 2.03)/1.93 (1.87 to 1.99)	1.98 (1.93 to 2.02)/1.93 (1.89 to 1.98)	.88
Occipital	2.05 (1.97 to 2.13)/2.06 (1.98 to 2.14)	2.06 (2.00 to 2.11)/2.07 (2.01 to 2.13)	.83
Global	2.10 (2.03 to 2.17)/2.07 (2.01 to 2.14)	2.08 (2.03 to 2.13)/2.06 (2.01 to 2.10)	.63
**Sulcal depth, mm**			
Frontal	3.29 (3.18 to 3.40)/3.19 (3.07 to 3.30)	3.18 (3.11 to 3.26)/3.08 (3.00 to 3.16)	.05
Parietal	4.54 (4.40 to 4.68)/4.48 (4.33 to 4.62)	4.33 (4.24 to 4.43)/4.27 (4.17 to 4.37)	.003
Temporal	3.09 (2.97 to 3.20)/3.02 (2.91 to 3.14)	3.07 (2.99 to 3.14)/3.00 (2.92 to 3.08)	.75
Occipital	3.15 (3.02 to 3.28)/3.16 (3.03 to 3.30)	3.09 (3.00 to 3.19)/3.10 (3.01 to 3.20)	.45
Global	3.55 (3.45 to 3.65)/3.47 (3.37 to 3.57)	3.45 (3.38 to 3.52)/3.37 (3.30 to 3.44)	.05
**Surface area, mm^2**			
Frontal	11 223 (10 682 to 11 765)/10 753 (10 200 to 11 306)	10 193 (9816 to 10 571)/9724 (9337 to 10 110)	<.001
Parietal	7325 (6926 to 7724)/7135 (6728 to 7542)	6834 (6554 to 7115)/6645 (6359 to 6930)	.01
Temporal	6525 (6212 to 6839)/6379 (6059 to 6699)	6124 (5903 to 6344)/5977 (5753 to 6202)	.01
Occipital	3861 (3612 to 4109)/3738 (3485 to 3992)	3636 (3463 to 3809)/3513 (3336 to 3691)	.05
Global	28 931 (27 556 to 30 306)/28 066 (26 662 to 29 469)	26 817 (25 860 to 27 775)/25 952 (24 973 to 26 931)	.003

[a]Results were derived from analysis of covariance, controlling for postmenstrual age at magnetic resonance imaging scan, sex, birth weight, maternal age, maternal smoking status, and maternal educational level.
[b]Adjusted for multiple testing based on the false discovery rate according to the Benjamini-Hochberg method. Adjusted $P < .05$ was considered statistically significant.

© 2026 Wu Y et al. *JAMA Network Open*.

eTable 4. Sex-Specific Comparison of Cerebral Cortical Folding in Newborns Antenatally Exposed to Different Opioid Types

Measure	Newborn group, least-squares mean (95% CI), male/female[a]		
	Nonexposed (n = 95)	Methadone exposed (n = 37)	Buprenorphine exposed (n = 108)
**Local gyrification index**			
Frontal	1.92 (1.86-1.97)/1.90 (1.84-1.95)	1.85 (1.79-1.90)/1.82 (1.76-1.88)	1.90 (1.86-1.94)/1.87 (1.83-1.91)
Parietal	2.48 (2.38-2.58)/2.44 (2.34-2.54)	2.40 (2.30-2.50)/2.36 (2.26-2.46)	2.47 (2.40-2.54)/2.43 (2.36-2.50)
Temporal	1.95 (1.89-2.02)/1.91 (1.85-1.98)	1.95 (1.89-2.02)/1.91 (1.84-1.97)	1.98 (1.93-2.03)/1.94 (1.89-1.98)
Occipital	2.04 (1.96-2.13)/2.05 (1.96-2.13)	2.03 (1.95-2.12)/2.03 (1.95-2.12)	2.06 (2.00-2.11)/2.06 (2.00-2.12)
Global	2.09 (2.02-2.15)/2.06 (1.99-2.12)	2.04 (1.97-2.11)/2.01 (1.94-2.08)	2.09 (2.04-2.13)/2.06 (2.01-2.11)
**Sulcal depth, mm**			
Frontal	3.28 (3.17-3.40)/3.18 (3.06-3.29)	3.16 (3.05-3.28)/]3.06 (2.94-3.18)	3.18 (3.10-3.26)/3.07 (2.99-3.16)
Parietal	4.50 (4.36-4.65)/4.44 (4.29-4.59)	4.30 (4.15-4.44)/4.24 (4.09-4.39)[b]	4.34 (4.24-4.45)/4.28 (4.18-4.39)[b]
Temporal	3.05 (2.93-3.17)/2.99 (2.87-3.11)	3.05 (2.94-3.17)/2.99 (2.87-3.12)	3.07 (2.98-3.15)/3.01 (2.92-3.09)
Occipital	3.18 (3.04-3.31)/3.18 (3.05-3.32)	3.08 (2.94-3.22)/3.09 (2.95-3.22)	3.08 (2.99-3.18)/3.09 (2.99-3.19)
Global	3.53 (3.43-3.63)/3.45 (3.35-3.55)	3.44 (3.33-3.54)/3.36 (3.25-3.46)	3.45 (3.38-3.53)/3.37 (3.30-3.45)
**Surface area, mm^2**			
Frontal	11 113 (10 580-11 646)/10 627 (10 087-11 167)	9725 (9183-10 268)/9239 (8686-9793)[c]	10 223 (9839-10 606)/(9341-10 132)[d]
Parietal	7221 (6819-7624)/7045 (6638-7452)	6565 (6155-6975)/6389 (5972-6806)[b]	6844 (6552-7136)/6668 (6369-6967)
Temporal	6481 (6167-6795)/6321 (6003-6639)	6005 (5683-6327)/5845 (5518-6172)[b]	6128 (5900-6356)/5968 (5734-6202)[b]
Occipital	3809 (3553-4065)/3699 (3440-3958)	3498 (3238-3758)/3388 (3122-3653)	3645 (3462-3829)/3535 (3346-3725)
Global	28 615 (27 245-29 986)/27 754 (26 366-29 141)	25 704 (24 310-27 098)/24 842 (23 422-26 262)[d]	26 909 (25 924-27 894)/26 047 (25 033-27 061)[b]

[a]Results were derived from analysis of covariance, controlling for postmenstrual age at magnetic resonance imaging scan, sex, birth weight, maternal age, maternal smoking status, and maternal educational level. Pairwise comparisons were performed among nonexposed, methadone-exposed, and buprenorphine-exposed groups in the overall sample. Adjusting for multiple testing was based on the false discovery rate according to the Benjamini-Hochberg method. Adjusted $P < .05$ was considered statistically significant.
[b]Significantly different from nonexposed controls (adjusted $P < .05$).
[c]Significantly different from nonexposed controls (adjusted $P < .001$).
[d]Significantly different from nonexposed controls (adjusted $P < .01$).

© 2026 Wu Y et al. *JAMA Network Open*.

eTable 5. Sex-Specific Comparison of Cerebral Sulcal Depth and Surface Area Among Newborns Exposed to Opioids Only or Opioids Plus Additional Substances and Nonexposed Controls

Measure	Newborn group, least-squares mean (95% CI), male/female[a]		
	Nonexposed (n = 95)	Opioids only (n = 45)	Opioids plus other substances (n = 119)[b]
**Sulcal depth, mm**			
Frontal	3.30 (3.19-3.41)/3.19 (3.08-3.31)	3.26 (3.15-3.37)/3.15 (3.04-3.26)	3.16 (3.08-3.24)/3.06 (2.98-3.14)[c]
Parietal	4.55 (4.41-4.69)/4.48 (4.34-4.62)	4.37 (4.23-4.51)/4.30 (4.16-4.44)[c]	4.32 (4.22-4.43)/4.25 (4.15-4.36)[d]
Temporal	3.10 (2.99-3.21)/3.03 (2.92-3.15)	3.13 (3.02-3.25)/3.07 (2.96-3.18)	3.05 (2.96-3.13)/2.98 (2.90-3.06)
Occipital	3.15 (3.02-3.28)/3.16 (3.02-3.29)	3.07 (2.93-3.20)/3.08 (2.95-3.21)	3.10 (3.01-3.20)/3.11 (3.01-3.21)
Global	3.56 (3.46-3.66)/3.48 (3.38-3.58)	3.51 (3.41-3.61)/3.42 (3.33-3.52)	3.44 (3.37-3.51)/3.35 (3.28-3.43)[c]
**Surface area, mm^2**			
Frontal	11 218 (10 671-11 765)/10 749 (10 193-11 306)	10 161 (9612-10 710)/9693 (9149-10 237)[d]	10 203 (9807-10 598)/9734 (9325-10 143)[d]
Parietal	7341 (6938-7744)/7147 (6738-7556)	6927 (6522-7331)/6733 (6334-7133)	6807 (6513-7101)/6613 (6312-6915)[c]
Temporal	6550 (6235-6866)/6398 (6077-6718)	6269 (5950-6588)/6117 (5802-6432)	6081 (5851-6311)/5928 (5691-6166)[d]
Occipital	3873 (3623-4124)/3748 (3493-4003)	3707 (3456-3959)/3582 (3333-3831)	3615 (3434-3796)/3490 (3302-3677)[b]
Global	28 957 (27 567-30 346)/28 085 (26 672-29 498)	26 965 (25 572-28 358)/26 094 (24 715-27 472)[c]	26 775 (25 772-27 777)/25 903 (24 868-26 939)[d]

[a]Results were derived from analysis of covariance, controlling for postmenstrual age at magnetic resonance imaging scan, sex, birth weight, maternal age, maternal smoking status, and maternal educational level. Pairwise comparisons were performed among nonexposed newborns, those exposed to opioids only, and those exposed to polysubstances in the overall sample. Adjusting for multiple testing was based on the false discovery rate according to the Benjamini-Hochberg method. Adjusted *P* < .05 was considered statistically significant.
[b]Other substances include cocaine, benzodiazepines, selective serotonin reuptake inhibitors, amphetamines, gabapentin, psychostimulants, muscle relaxants, tetrahydrocannabinol, and antipsychotics.
[c]Significantly different from nonexposed controls (adjusted *P* < .05).
[d]Significantly different from nonexposed controls (adjusted *P* < .01).

© 2026 Wu Y et al. *JAMA Network Open*.
