# Supplementary material for: Antenatal Opioid Exposure and Cerebral Cortical Maturation in Newborns
Source: JAMA Netw Open. 2026 May 22;9(5):e2614115. doi: 10.1001/jamanetworkopen.2026.14115 (PMC13197868; doi:10.1001/jamanetworkopen.2026.14115)
Supplement: Supplement 2. — Data Sharing Statement [file jamanetwopen-e2614115-s002.pdf]

Data Sharing Statement

Wu. Antenatal Opioid Exposure and Cerebral Cortical Maturation in Newborns. *JAMA Netw Open*. Published May 22, 2026. doi:10.1001/jamanetworkopen.2026.14115

Data
Data available: Yes
Data types: Deidentified participant data
How to access data: The clinical data will be submitted to NICHD Data and Specimen Hub (DASH) and the Helping to End Addiction Long-term (HEAL) Data Sharing Platform. The MRI data will be shared with OpenNeuro as per HEAL Initiative Data Sharing requirements.
When available: beginning date: 06-30-2027

Supporting Documents
Document types: None

Additional Information
Who can access the data: anyone
Types of analyses: for any purpose
Mechanisms of data availability: public access database
